# The effects of lymph node status on predicting outcome in ER+ /HER2- tamoxifen treated breast cancer patients using gene signatures

**DOI:** 10.1186/s12885-016-2501-0

**Published:** 2016-07-28

**Authors:** Jessica G. Cockburn, Robin M. Hallett, Amy E. Gillgrass, Kay N. Dias, T. Whelan, M. N. Levine, John A. Hassell, Anita Bane

**Affiliations:** 1 Department of Oncology, Juravinski Hospital and Cancer Centre, Hamilton, Canada; 2 Department of Biochemistry and Biomedical Sciences, Centre for Functional Genomics, McMaster University, Hamilton, Canada; 3 Department of Pathology, Juravinski Hospital and Cancer Centre, Hamilton, Canada

**Keywords:** Breast cancer, Lymph node status, Gene signature, Estrogen receptor, Prognosis, Oncotype DX, Prosigna

## Abstract

**Background:**

Lymph node (LN) status is the most important prognostic variable used to guide ER positive (+) breast cancer treatment. While a positive nodal status is traditionally associated with a poor prognosis, a subset of these patients respond well to treatment and achieve long-term survival. Several gene signatures have been established as a means of predicting outcome of breast cancer patients, but the development and indication for use of these assays varies. Here we compare the capacity of two approved gene signatures and a third novel signature to predict outcome in distinct LN negative (-) and LN+ populations. We also examine biological differences between tumours associated with LN- and LN+ disease.

**Methods:**

Gene expression data from publically available data sets was used to compare the ability of Oncotype DX and Prosigna to predict Distant Metastasis Free Survival (DMFS) using an *in silico* platform. A novel gene signature (Ellen) was developed by including patients with both LN- and LN+ disease and using Prediction Analysis of Microarrays (PAM) software. Gene Set Enrichment Analysis (GSEA) was used to determine biological pathways associated with patient outcome in both LN- and LN+ tumors.

**Results:**

The Oncotype DX gene signature, which only used LN- patients during development, significantly predicted outcome in LN- patients, but not LN+ patients. The Prosigna gene signature, which included both LN- and LN+ patients during development, predicted outcome in both LN- and LN+ patient groups. Ellen was also able to predict outcome in both LN- and LN+ patient groups. GSEA suggested that epigenetic modification may be related to poor outcome in LN- disease, whereas immune response may be related to good outcome in LN+ disease.

**Conclusions:**

We demonstrate the importance of incorporating lymph node status during the development of prognostic gene signatures. Ellen may be a useful tool to predict outcome of patients regardless of lymph node status, or for those with unknown lymph node status. Finally we present candidate biological processes, unique to LN- and LN+ disease, that may indicate risk of relapse.

**Electronic supplementary material:**

The online version of this article (doi:10.1186/s12885-016-2501-0) contains supplementary material, which is available to authorized users.

## Background

Axillary lymph node (LN) status is the most important prognostic variable in the management of patients with primary estrogen receptor positive (ER+) breast cancer, which accounts for the majority of diagnosed cases. Node positive breast cancer patients have been shown to have a worse prognosis than those with node negative disease. These observations have led, in part, to the development of a Tumour Nodal Metastases (TNM) staging system that incorporates tumour size, nodal involvement, including the absolute number of involved nodes, and the presence or absence of systemic metastases into an incremental staging system [1, 2]. Each stage of disease has specific survival characteristics and is thought to represent the natural progression of a tumour, from its origins in the breast to its metastasis through the lymphatic system to regional lymph nodes and ultimately through the circulatory system to distant sites. Clinicians use the TNM staging system to guide the management of breast cancer patients. Most breast cancer patients with involved axillary lymph nodes, in the absence of significant co-morbidities, are currently offered adjuvant systemic chemotherapy [3, 4].

However, the biological significance of nodal metastases is poorly understood. It is hypothesised that involvement of axillary lymph nodes is an indicator of tumour chronology such that the longer a tumour has been growing in the breast the more likely it is to metastasize to regional axillary nodes. Furthermore, it is thought that breast cancers first metastasize to these nodes and then secondarily to other sites [5, 6]. In support of this hypothesis, there is an established correlation between larger tumour size and lymph node involvement; indeed more timely intervention and resection of smaller primary tumours is associated with a reduced incidence of spread to regional lymph nodes [7]. More importantly, the absence of lymph node involvement is significantly associated with a better prognosis.

An alternative hypothesis suggests that some metastatic tumours avoid the lymphatic system, and instead spread primarily through the circulatory system [8, 9]. The evidence for this theory stems from the knowledge that 30 % of patients who are lymph node negative (LN-) at diagnosis will eventually succumb to metastatic breast disease, even after optimal treatment [10]. Conversely, there is a subset of patients who present with lymph node positive (LN+) disease that never develop distant recurrence, even in the absence of adjuvant treatment [9, 11]. It is likely that the biology of a primary tumour at diagnosis contributes to whether it remains at the primary site, spreads to regional lymph nodes, or metastasizes to distant sites via lymph node spread or through the vascular circulation. It is increasingly recognised that clinical pathological factors alone are limited in their ability to predict who will develop recurrent cancer or respond to treatment. To this end, a number of genomic signatures have been developed which have shown to be both prognostic (predict risk of distant recurrence) and predictive (predict response to chemotherapy) [12, 13]. It is thought that these signatures detect biological differences in primary tumours indicative of whether a tumour is likely to metastasize.

Here, we explore the relationship between stage and tumour biology to outcome in ER+ breast cancer, in the context of prognostic gene signatures, namely Oncotype DX and Prosigna [14–17]. Specifically, we compared the capacity of Oncotype DX, developed exclusively on and for LN negative (LN-) ER+ patients [17], and Prosigna, developed on all clinical subtypes of breast cancer including those with and without lymph involvement [18], for their capacity to predict outcome in patients with ER+/LN- and ER+/LN+ tumours. Furthermore, we examine the biological pathways represented in patient tumours with and without LN involvement that have good survival versus those that have developed systemic metastases. Finally, using this knowledge, a novel prognostic gene signature, called ‘Ellen’ was developed *in silico* for both LN+ and LN- ER+ breast cancer.

## Methods

### Patients and samples

All data was publicly available and downloaded from the Gene Expression Omnibus (GEO), NCBI [19] (http://ncbi.nlm.nih.gov/geo). Three independent experimental cohorts, GSE17705 [20] and GSE6532 [21] (which comprises 2 separate cohorts), were used for discovery and training and are briefly described in Table 1. Patients in all three cohorts were known to have ER+ tumours, were treated with surgical excision of the primary tumour and axillary dissection followed by 5 years of adjuvant tamoxifen. Limited pathological information is available for each sample, but ER and LN status is provided. The development of distant metastases was recorded over 10-years of clinical follow-up and reported as distant metastases free survival (DMFS). DMFS rates for LN- and LN+ patient subgroups were also reported. Patients with HER2 positive tumours were removed from all cohorts, as HER2 is known to be a poor prognostic variable for both LN+ and LN- tumours. Furthermore, in clinical practice patients with HER2+ ER+ tumours of 1 cm or more commonly receive adjuvant chemotherapy and Herceptin. A tumour was considered HER2 positive if either of the two *HER2* probes on the Affymetrix chip were overexpressed as calculated using previously published methods [22].

**Table 1:** Summary of GEO cohort characteristics

	GSE17705	GSE6532-C
*n*	230	132
LN Positive	91 59 % DMFS at 10 years	89 70 % DMFS at 10 years
LN Negative	139 85 % DMFS at 10 years	43 75 % DMFS at 10 years
Age	NR	61
Age Range	NR	40–88
Distant Metastasis at 10 years (*n*)	62	33
Overall 10 year DMFS	58.7 %	71.1 %
Location	MD Anderson, US	Guy’s Hosptial, UK, John Radcliffe Hospital, UK & Uppsala University Hospital, Sweden
Submission Group	Hatzis, Nuvera Biosciences, Woburn Mass	Loi et al., Institut Jules Bordet, Belgium
Affymetrix Chip	U133.A	U133.A & U133.2

NR-Not Reported

GSE17705 was used as a training cohort for feature discovery in the generation of the Ellen signature and comprises Affymetrix U133A chip microarray expression data from 230 ER+/HER2- primary breast cancers, ~40 % of which were LN+. Two additional independent cohorts, GSE6532-A and GSE6532-2, were combined (GSE6532-C) and used to examine the Oncotype DX and Prosigna assays, and to validate the Ellen signature derived from the training cohort. The GSE6532-C cohort contained Affymetrix U133A and U133 Plus 2.0 microarray expression data from 132 ER+/HER2- primary tumours, ~67 % of the patients were lymph node positive. Specific demographic information for GSE17705 and GSE6532 can be found on the GEO website and in previously published reports [19, 20].

#### Data preparation

To extract the data from these cohorts, the raw intensity files (.CEL) comprising each dataset were downloaded and normalized using the Robust Multichip Algorithm (RMA) [23, 24] to generate a single intensity value for each probeset, using GenePattern (Broad Institute, Cambridge, Massachusetts). This preprocessing method has also been shown to yield concordance with qRT-PCR values and has been used in similar studies [24, 25]. Intensity was standardized using a Z score, where probe intensity was averaged among all samples and subtracted from the probe intensity from a single sample, which was then divided by the standard deviation of the probe intensities. Several other peer reviewed articles refer to a similar method to mimic qRT-PCR based assays using microarray gene expression data [25].

### Oncotype DX analysis

To simulate the Oncotype DX assay, only probesets corresponding to the prognostic genes comprising the Oncotype DX gene list were selected. The Oncotype DX recurrence score (RS) is calculated by taking a modified weighted average for each functionally distinct group of genes, which were then combined [17]. The use of *ACTB*, *GAPDH*, and *TFRC* transcripts was excluded as data had been initially normalized using RMA. It is important to note that the range of recurrence scores differs between qRT-PCR (quantitative Real Time-Polymerase Chain Reaction) (RS are greater than 0) and expression microarray platforms (RS normally distributed around zero), as qRT-PCR data distribution is cumulative and microarray data is continuous.

### Prosigna analysis

To simulate the Prosigna assay, expression values from only the available (*n* = 45) Affymetrix probe sets corresponding to the 50 Prosigna genes were used. Six genes (ANL*N, CDCA1, CXXC5, FOXC1, TMEM45B, UBE2T*) from the Prosigna assay, representing both pro- and anti-tumour functions were excluded from the analysis because probesets representing these genes were not represented on the Affymetrix chips. Standardized expression microarray values were used, in place of Nanostring nCounter expression data. The risk of recurrence (ROR) score was calculated using the Spearman correlation of prognostic gene expression to predetermined coefficients relating to the expected expression of each gene based on the intrinsic molecular subtypes as described [18].

### Signature performance

Cox Proportional Hazards Regression analysis was used to determine the non-parametric association of continuous signature scores to patient outcome over time. The Cox PH package in R (R Foundation for Statistical Computing, Vienna, Austria) was used to calculate Concordance (C), hazard ratio (HR), p values, and confidence intervals (CI) for each signature. Analysis of signatures was simultaneously performed using all eligible tumours irrespective of patient outcome. Signature performance was compared using statistical variables alone and in the absence of prior knowledge to signature performance in the test cohort. Significant differences between outcome groups were determined by statistical alpha values being less than or equal to 0.05 for each test or the CI range excluding 1, as appropriate. Kaplan-Meier survival curves were generated using the median cut-point for each signature scores to visually represent outcome of patients at high versus low risk of distant metastasis.

### Gene set enrichment analysis

Gene set enrichment analysis (GSEA) from Gene Pattern (Broad Institute, Cambridge, Massachusetts), was used to evaluate the biological mechanisms represented by sets of genes associated with distant metastasis free survival (DMFS) in patients with ER+ breast cancer, as previously described [26, 27]. Briefly, LN- and LN+ patient groups were classed by outcome (presence or absence of metastases) and associated Affymetrix data was used to enrich for gene sets. The GSEA algorithm ranks all genes by expression level in either class of samples. It then compares the pattern and frequency of gene expression in each class to previously published gene lists using an iterative approach to find the most related gene sets. An enrichment score (ES) is calculated for each gene set in each cohort, which can then be extrapolated to biological significance. Reported functions of individual genes are from the Gene Ontology Consortium (Release date April 2016, http://geneontology.org) [28].

### Development and validation of the Ellen signature

#### Identification of prognostic genes

Prediction Analysis of Microarrays (PAM) [29] was used for feature selection and 10-fold cross-validation was used to estimate the optimal number of features (genes) to comprise the gene signature. DMFS was used as the clinical end-point.

#### Validation of gene signature

To calculate a final prognostic index, gene Z scores were averaged by outcome association and then subtracted such that the average of poor outcome probesets was subtracted from the average of good outcome probesets, resulting in positive correlation to DMFS. Again, 10 year DMFS was used as the clinical endpoint and Cox PH Regression, C, and HRs were used to evaluate signature performance.

## Results

### *In silico* validation

We independently verified the ability of Oncotype DX to predict recurrence in LN- patients in the training cohort using microarray expression data to ensure the validity of our *in silico* strategy (*p* <1.2x10^2^, HR: 3.58) (Table 2). Similar *in silico* approaches have previously been used to replicate gene signatures, including Oncotype DX and Prosigna [30–32]*.*

**Table 2:** Oncotype DX validation on GSE17705

	HR	*p* value	CI upper	CI lower
Combined Patients	1.74	5.5E-02	0.99	3.07
LN- Patients	3.58	1.2E-02	1.38	9.27
LN+ Patients	1.16	6.8E-01	0.57	2.34

### Signature comparison

**Fig. 1:**
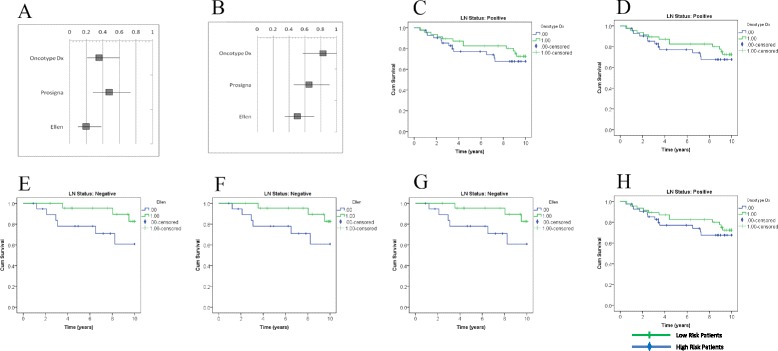
Performance of Gene Signatures. Comparison of hazard ratios (HR) with 95 % confidence intervals from Oncotype DX, Prosigna, and Ellen. Signature performance on LN- patients (**a**) and LN+ patients (**b**) exclusively. Cumulative survival (Cum Survival) over 10 years of follow-up is demonstrated using Kaplan-Meier survival curves. Individual curves represent median cut-points of Oncotype DX (**c** and **d**), Prosigna (**e** and **f**), and Ellen (**g** and **h**) signatures that are shown for by LN- (**c**, **e**, and **g**) and LN+ (**d**, **f**, and **h**) patients respectively. The curves represent patients at high or low risk of metastasis

We examined the performance of the Oncotype DX and Prosigna gene signatures on transcript profiles of breast cancer patients with either LN- or LN+ disease. To do so, the Oncotype DX algorithm was replicated *in silico* using Affymetrix gene expression data as described above. We subsequently tested the prognostic ability of the simulated algorithm on ER+ tumours from LN + and LN- patients. As expected, the simulated Oncotype DX algorithm was able to significantly predict outcome for ER+ LN- patients (*p* <1.26x10^4^, HR: 0.36, C:0.78) (Fig. 1 and Table 3) which confirms its prognostic capacity in this group of patients. We also used the modified Oncotype DX algorithm, to predict outcome of ER+ LN+ patients. Oncotype DX was unable to predict risk of recurrence for ER+ LN+ patients from GSE6532-C (*p* > 0.30) (Fig. 1 & Table 3).

**Table 3:** Oncotype DX, Prosigna, and Ellen performance

	LN- Patients		LN+ Patients	
	*p* value	Concordance	*p* value	Concordance
Oncotype DX	1.26E-04	0.78	3.06E-01	0.58
Prosigna	8.07E-04	0.79	1.34E-02	0.62
Ellen	1.27E-06	0.85	1.74E-04	0.71

We subsequently simulated the Prosigna gene assay *in silico* using Affymetrix gene expression data, as described in the methods. As expected, the simulated Prosigna signature was able to significantly predict outcome for ER+ LN- patients (*p* <8.07x10^4^, HR: 0.48, C:0.79) (Fig. 1 and Table 3), as well as in ER+ LN+ patients (*p* <1.34x10^2^, HR: 0.65, C: 0.62) (Fig. 1 and Table 3).

We then developed an independent signature, known as “Ellen”, using both LN- and LN+ patients from the training cohort, and demonstrated that it was able to more significantly predict outcome of LN- and LN+ cohorts than either the Oncotype DX or Prosigna gene signatures. For LN- patients, Ellen scores were associated with the ability to predict risk of relapse with a concordance of 0.85 and hazard ratio of 0.20 (*p* <1.27 × 10^6^) (Fig. 1 and Table 3). Similarly, for LN+ patients Ellen score was able to predict risk of distant metastasis with a concordance of 0.71 and hazard ratio of 0.50 (*p* <1.74 × 10^4^).

**Fig. 2:**
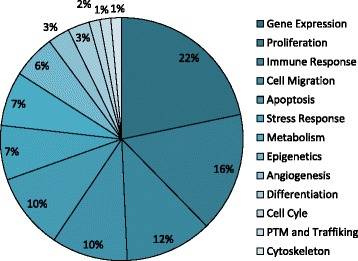
Biological pathways. Graphical distribution of biological pathways represented within the Ellen gene signature, as determined by number of genes associated with each pathway

The Ellen gene signature comprises 57 genes; expression of 33 of these genes is associated with a low risk of distant metastasis whereas expression of 24 is associated with high risk (Table 4). The biological processes of the genes present in all three signatures (Ellen, Oncotype DX and Prosigna) were functionally annotated using the Gene Ontology Consortium (Fig. 2 and Table 4). All three signatures included genes with functions related to gene expression, proliferation, immune response, cell migration, cell cycle, and post translational modification (PTM) and trafficking. Ellen and Prosigna each contained genes that represented unique biological processes; namely epigenetic and angiogenic processes for Ellen and DNA repair and replication processes for Prosigna (Table 5). Direct comparison of gene lists showed that there are 11 overlapping genes between Oncotype DX and Prosigna (BAG1, BCL2, BIRC5, CCNB1, ERBB2, ESR1, GRB7, MKI67, MMP11, MYBL2, PGR) and no additional overlapping genes between Ellen and either of the other two signatures.

**Table 4:** Number of Ellen genes associated with different biological pathways

	Low Risk of Metastasis Genes	High Risk of Metastasis Genes	Total
Gene Expression	13	2	15
	EGR1, FOS, JUN, NAT10, RPL11, ZFP36, EEF2, LITAF, POLR2E, POLR3E, RPLP2, RPS15, RPS23	RPL38, RPS11	
Proliferation	5	6	11
	KIDINS220, PIK3R1, ZFP36L2, CDIPT, CXCL12	JTB, SERPINB3, NUCKS1, SNRPE, SPDEF, TXN	
Immune Response	5	3	8
	FOS, CXCL12, HLA-DPA1, JAK1, PCBP2	FKBP4, MTDH, NUCKS1	
Cell Migration	3	4	7
	SPTBN1, CYFIP1, CXCL12	S100P, ARF6, CSTA, NUCKS1	
Apoptosis	6	1	7
	JUN, SGK1, LITAF, TNFRSF10B, GLTSCR2, ITM2B	S100G(-)	
Stress Response	5	0	5
	DUSP1, ABAT, CIRBP, GLTSCR2, GPX4		
Metabolism	1	4	5
	GPX4	FLOT1, SQLE, COX5B, GPR172A	
Epigenetics	2	2	4
	NCOR1, SMARCA2	NAT10, H3F3A	
Angiogenesis	0	2	2
		ACTC1, MB	
Differentiation	2	0	2
	DPYSL2, RAI2		
Cell Cycle	0	1	1
		SFN	
PTM and Trafficking	1	0	1
	DUSP1		
Cytoskeleton	0	1	1
		KRT10

**Table 5:** Comparison of biological processes associated with each gene signature

		Signature		
	Biological Pathway	Ellen	Oncotype DX	Prosigna
Common	Gene expression	15	2	5
	Proliferation	11	4	15
	Immune response	8	3	1
	Cell migration	7	4	9
	Cell cycle	1	4	12
	PTM and trafficking	1	2	4
Unique	Epigenetics	4	0	0
	Angiogenesis	2	0	0
	DNA repair	0	0	4
	DNA replication	0	0	3
Other	Apoptosis	7	0	1
	Metabolism	5	0	2
	Stress response	5	0	2
	Differentiation	2	0	2
	Cytoskeleton	1	0	5
	Intracellular signalling	0	3	6
	Survival	0	2	2
	Drug metabolism	0	1	1

### Biological differences between LN status and outcome

Gene Set Enrichment Analysis (GSEA) was used to identify biological processes potentially related to outcome in ER+ tumours with and without lymph node involvement. The GSEA algorithm was performed independently on LN+ and LN- samples, using systemic recurrence as the phenotypic class variable. Based on these findings, biological pathways that are related to outcome in LN- (Table 6) and LN+ (Table 7) patients groups were identified. Additional information pertaining to specific overlapping genes and statistical parameters is available in the Additional file 1. A number of cancer-related pathways were enriched in each subgroup of patient samples, including proliferation, epithelial-mesenchymal transition (EMT), epigenetic modification, and immunity [33]. Poor outcome LN- patient tumours were enriched for proliferation, growth factor signalling and epigenetic modification gene sets (Table 6). Whereas, poor outcome LN+ patient tumours were enriched for gene sets associated with EMT, migration, differentiation, and apoptosis. The tumours from patients with good survival, both LN- and LN+, were enriched for immune response gene sets. This was particularly evident for patients with LN+ disease where 6 of the top 10 gene sets, associated with good outcome were comprised of 649 immune response related genes (Table 7).

**Table 6:** Gene sets enriched in lymph node negative patients

LN-	Gene Set Name	Inferred Biological Activity	Genes Represented	ES
Long Term Remission	SHEN_SMARCA2_TARGETS_UP	Epigenetic	347	0.65
	SCHUETZ_BREAST_CANCER_DUCTAL_INVASIVE_UP		321	0.60
	BOQUEST_STEM_CELL_UP	Stemness	239	0.57
	ANASTASSIOU_CANCER_MESENCHYMAL_TRANSITION_SIGNATURE	EMT	60	0.71
	SMID_BREAST_CANCER_LUMINAL_A_UP		78	0.67
	PICCALUGA_ANGIOIMMUNOBLASTIC_LYMPHOMA_UP	Immune	171	0.58
	TURASHVILI_BREAST_DUCTAL_CARCINOMA_VS_LOBULAR_NORMAL_DN		52	0.71
	DACOSTA_UV_RESPONSE_VIA_ERCC3_COMMON_DN	Radiation	409	0.52
	FLECHNER_BIOPSY_KIDNEY_TRANSPLANT_OK_VS_DONOR_UP	Immune	480	0.51
	REN_ALVEOLAR_RHABDOMYOSARCOMA_DN	PAX3-FOXO1 down	386	0.52
Distant Metastasis	SHEN_SMARCA2_TARGETS_DN	Epigenetic	312	−0.54
	RICKMAN_HEAD_AND_NECK_CANCER_F		47	−0.72
	SOTIRIOU_BREAST_CANCER_GRADE_1_VS_3_UP		130	−0.54
	ROSTY_CERVICAL_CANCER_PROLIFERATION_CLUSTER	Proliferation	124	−0.51
	KEGG_OLFACTORY_TRANSDUCTION	Signalling	66	−0.58
	REACTOME_STRIATED_MUSCLE_CONTRACTION		27	−0.70
	KUNINGER_IGF1_VS_PDGFB_TARGETS_UP	IGF	58	−0.58
	XU_HGF_TARGETS_REPRESSED_BY_AKT1_DN	AKT/HGF	81	−0.51
	MIKKELSEN_MEF_ICP_WITH_H3K27ME3	Epigenetic	118	−0.48
	MIKKELSEN_MCV6_LCP_WITH_H3K27ME3	Epigenetic	15	−0.76

**Table 7:** Gene Sets enriched in lymph node positive patients

LN+	Gene Set Name	Inferred Biological Activity	Genes Represented	ES
Long Term Remission	SMID_BREAST_CANCER_NORMAL_LIKE_UP		420	0.65
	WIELAND_UP_BY_HBV_INFECTION	Immune	91	0.77
	WALLACE_PROSTATE_CANCER_RACE_UP	Increased Risk	259	0.66
	MCLACHLAN_DENTAL_CARIES_UP	Immune	225	0.59
	KIM_LRRC3B_TARGETS	Immune	28	0.85
	FLECHNER_BIOPSY_KIDNEY_TRANSPLANT_REJECTED_VS_OK_UP	Immune	78	0.67
	VANTVEER_BREAST_CANCER_ESR1_DN	ER down	197	0.59
	TURASHVILI_BREAST_DUCTAL_CARCINOMA_VS_DUCTAL_NORMAL_DN		144	0.60
	ICHIBA_GRAFT_VERSUS_HOST_DISEASE_D7_UP	Immune	89	0.66
	LEE_DIFFERENTIATING_T_LYMPHOCYTE	Immune	138	0.61
Distant Metastasis	NIKOLSKY_BREAST_CANCER_8Q12_Q22_AMPLICON		86	−0.68
	NIKOLSKY_BREAST_CANCER_8Q23_Q24_AMPLICON		83	−0.66
	ANASTASSIOU_CANCER_MESENCHYMAL_TRANSITION_SIGNATURE	EMT	60	−0.70
	TURASHVILI_BREAST_DUCTAL_CARCINOMA_VS_DUCTAL_NORMAL_UP		28	−0.65
	FARMER_BREAST_CANCER_CLUSTER_5		18	−0.74
	TURASHVILI_BREAST_LOBULAR_CARCINOMA_VS_DUCTAL_NORMAL_UP		52	−0.55
	MILI_PSEUDOPODIA_HAPTOTAXIS_UP	Migration	348	−0.41
	DING_LUNG_CANCER_EXPRESSION_BY_COPY_NUMBER	CNVs	81	−0.49
	IIZUKA_LIVER_CANCER_PROGRESSION_G2_G3_UP	Differentiation	24	−0.64
	HAMAI_APOPTOSIS_VIA_TRAIL_UP	Apoptosis	463	−0.38

## Discussion

Lymph node status is the most prognostic variable for determining outcome in patients with ER+ breast cancer. However, it is unknown whether lymph node involvement is simply an indication of tumour progression over time or whether a primary tumour’s ability to metastasize is pre-determined by tumour biology. Gene signatures are an attractive option to predict outcome and several have been validated for use on ER+ breast cancer patients. Oncotype DX is a prognostic (and predictive) gene signature developed and validated using ER+ LN- tumours exclusively, whereas the development of the Prosigna gene signature included LN+ tumour samples. We wanted to examine the performance of Oncotype DX and Prosigna on LN+ patients and hypothesized that if lymph node involvement is merely a function of tumour progression, then the signatures developed using LN- patient samples (Oncotype DX) should similarly be able to predict outcome for LN + patients.

The Oncotype DX signature was developed using weighted averages of 16 genes (excluding housekeeping genes) known to be associated with outcome in ER+ LN- breast cancer using a qRT-PCR platform [17]. This 21 gene signature has been validated and FDA approved for its ability to predict outcome in an independent cohort of ER+ LN- breast cancer patients [34, 35]. We simulated the Oncotype DX algorithm *in silico* using Affymetrix gene expression data and tested the prognostic ability of the simulated algorithm on ER+ tumours from LN+ and LN- patients. As expected, the simulated Oncotype DX algorithm was able to significantly predict outcome for ER+ LN- patients, confirming its prognostic capacity in this group of patients and supporting the validity of our *in silico* approach to assess Oncotype DX performance. Furthermore, the *in silico* approach we utilized has been used by others to compare gene expression data from different platforms including qRT-PCR and expression microarrays and to simulate gene signatures such as Oncotype DX and Prosigna [24, 29–31, 33–35].

In our *in silico* study, Oncotype DX was unable to significantly predict risk of recurrence for ER+ LN+ patients (Fig. 1 and Table 3), suggesting that a signature such as Oncotype DX, developed and validated on ER+ LN- patients, is not optimal for predicting outcome in ER+ LN+ patients. We cannot exclude the possibility that there is a subset of LN+ patients for whom Oncotype DX might be an appropriate prognostic assay, but further exploration in this area is needed. As such, there are several ongoing clinical trials, including SWOG S1007 and RxPONDER aimed at validating the prognostic utility of Oncotype DX for ER+ breast cancer patients with limited LN+ disease, the results from these studies are eagerly awaited [36, 37].

Prosigna was approved as a prognostic assay for distant metastasis-free survival for patients with ER+ disease with 0–3 positive lymph nodes. The 50 disease associated-genes comprising the Prosigna assay were derived from the intrinsic molecular subtype signatures discovered in 2000 [18, 38]; both LN- and LN+ breast cancer samples were used to develop and validate the Prosigna assay ([39], TransATAC and ABCSG8 clinical trials). The simulated Prosigna signature, described here was able to significantly predict outcome for ER+ LN- and LN+ patients separately. This suggests that including LN+ patient samples in signature development will improve signature performance when applied to LN+ patient tumour samples.

The Ellen signature, which was developed using both LN- and LN+ patients, was able to more significantly predict outcome of LN- and LN+ cohorts than either the Oncotype DX or Prosigna gene signatures. It is possible that the increased significance, concordance, and hazard ratios derived from the Ellen signature are related to it being both trained and validated using Affymetrix data and we recognize that our results need to be validated using an independent cohort of patients. Alternatively, the increased significance of Ellen could be reflective of the importance of the biological processes, represented by the signature genes, to outcome in ER+ breast cancer. As detailed in Table 5, Ellen, Oncotype DX, and Prosigna signatures each represent common biological processes including: gene expression, proliferation, immune response, cell migration, cell cycle, and PTM and Trafficking. However, genes related to angiogenesis and epigenetics are unique to Ellen. Both of these processes have been demonstrated to be important for outcome in ER+ breast cancer [6, 14, 33, 40–43]. Additional multivariable studies are being conducted, using an independent cohort of patients, to assess the relationship between these biological features and other clinical variables, including tumour size, grade, and histological subtype to validate the prognostic potential of Ellen.

Given that the three signatures examined performed with various levels of accuracy in LN+ and LN- patient populations, we were interested in exploring the biological processes that might be related to outcome in ER+ LN+ and LN- tumours separately, using GSEA. Patients with good outcome (irrespective of their original LN status) had tumours with expression profiles enriched for immune related genes (Tables 6 and 7). This was particularly striking for LN+ tumours where 6 of the 10 gene sets associated with good outcome were immune related. This enrichment of immune related gene sets may be indicative of immune cell infiltration in some tumours and suggests that a subset of ER+ breast cancer patients have a robust anti-tumour immune response and that this in turn may be associated with improved survival [39, 44, 45].

We examined the ontology of genes comprising the Ellen signature to determine whether their functions overlap with those identified using the GSEA and found that 11 % of the Ellen genes are related to immune response. This further supports an important role for immune response in ER+ tumours and the utility of the signature. For example, we found that CXCL12 and JAK1 are both more highly expressed in low risk tumours. It has been reported that increased expression of CXCL12 is a strong positive prognostic factor that correlates with disease free and overall survival in both ER+ and ER- tumours [46, 47]. JAK1 is a protein tyrosine kinase involved in the response to interferons; recently the closely related JAK2 family member was found to be associated with improved outcome in breast cancer [48]. In addition, the expression of HLA-DPA1, which is normally expressed on antigen presenting cells, may indicate the presence of immune infiltrate [49]. Overall, the presence of these immune related genes in low risk tumours indicates that immune response is an important factor in the progression of breast cancer.

Patients with poor outcome showed enrichment for different gene sets depending on whether their tumour was LN+ or LN- at diagnosis. For example, poor outcome LN- patient tumours were enriched for proliferation, growth factor signalling, and epigenetic modification gene sets, also represented by individual genes comprising the Ellen signature (Table 6). Proliferation in ER+ breast cancer is a poor prognostic factor and correlates with the Luminal B subtype [39]. Epigenetic modification is thought to have some role in tumour progression, as global hypermethylation of the tumour genome has been associated with poor outcome [50–52]. In addition there are several studies reporting that HDAC inhibitor usage may be useful as adjuvant chemotherapeutics in this high risk group [53, 54]. Whereas, patients with LN+ disease and poor outcome had tumours enriched for EMT and migration suggesting a migratory phenotype [9, 55].

Taken together, the different biological processes highlighted for LN- and LN+ groups may explain why gene signatures developed for one group would not necessarily be predictive of outcome in the other.

## Conclusion

In summary, we have shown that by comparing Oncotype DX and Prosigna with a novel gene signature, it is important to include patients with both LN+ and LN- status when developing prognostic gene signatures. Furthermore, we have identified candidate biological processes that imply how tumour biology can be related to outcome. This is particularly evident for LN+ tumours with good outcome, where there is enrichment in immune response gene expression, and for LN- tumours with poor outcome, where there is an enrichment for genes involved in epigenetic modification. We developed and characterized Ellen, a gene signature that is designed to be predictive of outcome for all patients with ER+ breast cancer without distant spread, using an unbiased gene selection process. The genes represented in this signature are similar to those whose pathways were found to be enriched using GSEA, further suggesting that Ellen would be suitable for use in a variety of biologically unique ER+ breast tumours. Work is currently underway to validate the performance of Ellen using an alternate platform and with additional independent cohorts. Further, the clinical information available for the training and validation cohorts was limited, so it is difficult to know whether there are other confounding variables. Ultimately, this study shows that gene expression of primary tumours can be informative about metastatic potential and can be distinguished between LN- and LN+ patients. In addition Ellen, once validated, would be able to provide prognostic information for patients with tumours accompanied by small lymph node metastasis, such as isolated tumour cells or micrometastases, those with incomplete lymph node dissections (ie sentinel node only), or those who have no lymph node information.

## Abbreviations

BC, breast cancer; C, concordance; CI, confidence interval; CoxPH, Cox proportional hazards; DMFS, distant metastasis free survival; EMT, epithelial mesenchymal transition; ER, estrogen receptor; ES, enrichment score; GEO, gene expression omnibus; GO, gene ontology; GSEA, gene set enrichment analysis; HR, hazard ratio; LN, lymph node; PAM, prediction analysis of microarrays; PTM, post translational modification; qRT-PCR, quantitative real time polymerase chain reaction; RMA, robust multichip algorithm; ROR, risk of recurrence; RS, recurrence score; TNM, tumour node metastasis

## Additional file

Additional file 1: (RAR 837 kb)
